# Characterization of humoral responses to soluble trimeric HIV gp140 from a clade A Ugandan field isolate

**DOI:** 10.1186/1479-5876-11-165

**Published:** 2013-07-08

**Authors:** Maria Luisa Visciano, Maria Tagliamonte, Guillaume Stewart-Jones, Leo Heyndrickx, Guido Vanham, Marianne Jansson, Anders Fomsgaard, Berit Grevstad, Meghna Ramaswamy, Franco M Buonaguro, Maria Lina Tornesello, Priscilla Biswas, Gabriella Scarlatti, Luigi Buonaguro

**Affiliations:** 1 Molecular Biology and Viral Oncogenesis Unit, Department of Experimental Oncology, Istituto Nazionale per lo Studio e la Cura dei Tumori “Fondazione Pascale” - IRCCS, Naples, Italy; 2 Human Immunology Unit, Weatherall Institute of Molecular Medicine, Oxford University, Oxford, UK; 3 Virology Unit, Institute of Tropical Medicine, Antwerp, Belgium; 4 Department of Laboratory Medicine, University of Lund, Lund, Sweden; 5 Statens Serum Institut, Copenhagen, Denmark; 6 National Institute for Biological Standards and Control, Hertfordshire, UK; 7 San Raffaele Scientific Institute, Milan, Italy

## Abstract

Trimeric soluble forms of HIV gp140 envelope glycoproteins represent one of the closest molecular structures compared to native spikes present on intact virus particles. Trimeric soluble gp140 have been generated by several groups and such molecules have been shown to induce antibodies with neutralizing activity against homologous and heterologous viruses. In the present study, we generated a recombinant trimeric soluble gp140, derived from a previously identified Ugandan A-clade HIV field isolate (gp140_94UG018_). Antibodies elicited in immunized rabbits show a broad binding pattern to HIV envelopes of different clades. An epitope mapping analysis reveals that, on average, the binding is mostly focused on the C1, C2, V3, V5 and C5 regions. Immune sera show neutralization activity to Tier 1 isolates of different clades, demonstrating cross clade neutralizing activity which needs to be further broadened by possible structural modifications of the clade A gp140_94UG018_. Our results provide a rationale for the design and evaluation of immunogens and the clade A gp140_94UG018_ shows promising characteristics for potential involvement in an effective HIV vaccine with broad activity.

## Introduction

Major challenges in the development of an HIV vaccine have been the design of immunogens able to induce a strong and sustained immunity with a broad and cross-clade neutralizing activity.

In the course of natural infection, although HIV-1 is highly effective in evading the immune surveillance [1-3], almost 20% of HIV-infected subjects are able to develop antibodies with a broad degree of neutralization activity, whose role in the disease control, however, is still debated (reviewed in [4]. Such evidences, indeed, suggest that native antigens are able to elicit such bnAbs antibodies. To date, a number of human bnAbs targeting the HIV envelope glycoprotein in its trimeric status have been isolated from HIV-1 infected subjects [5-14].

Trimeric envelope structures, either soluble or protruding from a membrane-surrounded particle, have been explored as vaccine models for eliciting broadly neutralizing antibodies (bnAbs) [15-19].

Indeed, the native and functional HIV-1 envelope glycoprotein (Env) complex is present on the virus surface as a trimer, each of the monomers made of non-covalently loosely associated gp120 surface and gp41 transmembrane glycoproteins [20-23]. However, recombinant soluble forms of fully cleaved and functional trimers, have been difficult to obtain for their high instability. On the other hand, the use of gp160 ectodomain (gp140) has led to the production of trimers that can mimic the native Env spike and have shown to be able to elicit neutralizing antibody responses in immunized animals [24-27]. These gp140 trimers can be further stabilized by introduction of specific modification in order to strenghten intra- as well as inter-molecular bindings (gp140_SOSIP_) [28,29].

We have recently used a similar strategy to present trimeric gp140 Env molecules on HIV Virus-Like Particles produced in both a transient baculovirus expression system [19] and a stably transfected insect cell line [18]. In particular, a gp120 Env molecule derived from a Ugandan HIV-1 isolate of the clade A (94UG018; GenBank accession number AF062521) [30,31] has previously been shown to induce high Ab titers with cross-clade neutralizing activity in immunized BALB/C mice [32-34] and non-human primates [35].

In the present study, the same gp140_94UG018_ presented on the surface of VLPs has been produced as recombinant soluble trimeric form of Env for evaluation in homologous prime-boost immunization schedules. An immunogenicity study has been performed in rabbits to evaluate the potency and broadness of specific humoral immune response as well as the mapping of the epitope recognition by the Abs elicited by such protein.

## Material and methods

### Protein immunogen

Recombinant gp140 protein was produced by transient transfection of the pLex-gp140_94UG018_ plasmid into adherent 293T cells grown in DMEM media supplemented with 10% FCS. Supernatants were collected after 48 hours and fresh media, containing 10% FCS was added to the cells for another 48 hours at which time point the media was collected again. Following centrifugation and filtration of the supernatant through a 0.22 μm filter, the protein was isolated from the pooled supernatants by initial capture using Talon IMAC chromatography, and elution with TBS with 250 mM imidazole followed by GNL Lectin (Vector Labs) capture and eluted with 1M methyl α-D mannopyranoside, 100 mM sodium acetate (pH 4.0) and finally gel filtration with a SD200 column equilibrated with PBS. The protein production and purification was performed using endotoxin-free materials and buffers. The protein was concentrated using a 10 kDa molecular weight cutoff protein concentrator to 1 mg/ml in PBS for immunization.

### Immunization protocol

Four female New Zealand white rabbits 6 to 8 weeks old (with a body weight of ~2 kg), were subcutaneously immunized with the trimeric Clade A gp140_94UG018_ in the presence of the adjuvant CAF01 [36]. Immunizations were performed at week 0, 2, 4 and 8 and each rabbit received 100μg/dose of immunogen. Blood was collected 3 days before the immunization protocol started (pre-immunization) and four (week 12) and six (week 14) weeks after the last immunization. Heat inactivated sera were stored at −80°C until used. All animal handlers were certified in laboratory animal science courses complying with the category B or C requirements of the Federation of European Laboratory Animal Science Association (FELASA). Animal experiments were performed according to the Animal Experimentation Act of Denmark and European Convention ETS 123 (Protection of Vertebrate Animals used for experimental and other scientific purposes).

### ELISA assays

#### Measurement of specific anti-HIV Env IgG antibodies in rabbit serum

The level of anti-envelope specific IgG antibodies in sera of immunized rabbits was determined by ELISA. Five antigens were used: gp140_94UG018_ (Clade A), gp120 _IIIB_ (Clade B), gp120 _W61D_ (Clade B), gp140 _ZM96_ (Clade C) or gp140 _UG037_ (Clade A). Briefly, 100 or 200 μL of each antigen at a final concentration of 1 μg/mL in PBS were directly coated on 96-well MICROTEST assay plates (Becton Dickinson) and plates were incubated overnight at 4°C. Five-fold dilutions (starting from 1:1000) of each rabbit serum were added to the wells and incubated 2 hrs at 37°C. After washing, 100 μL of goat anti-rabbit IgG horseradish peroxidase (HRP)-conjugated was added to each well at a concentration of 1 μg/mL. A TMB Ultra 1-step solution (Thermo Scientific) was used to develop the reactions which were stopped after 30 minutes with 100 μl/well of 2N H_2_SO_4_. Plates were read at 450 nm. Reactions were considered positive when the optical density (OD) measured by the ELISA was higher than the O.D. + 3SD of the same dilution of pre-immunization sera.

#### B-cell epitope mapping

B-cell epitope mapping was carried out using 20 mer overlapping synthetic peptides spanning the entire length of IIIB gp120 from C1 to C5 region. For each region 20 mer overlapping synthetic peptides were pooled or singularly coated at a final concentration of 1 μg/ml per each peptide directly on 96-well MICROTEST assay plates (Becton Dickinson) which were incubated overnight at 4°C. Each rabbit serum was added at a dilution of 1:1000 and incubated 2 hrs at 37°C against pools or single peptides. Each tested pool covered a region of gp120. Goat anti-rabbit IgG HRP-conjugated was added to each well at a concentration of 1 μg/mL and the same procedure as indicated above was followed.

#### Neutralization assay

IgG were purified from heat inactivated (1 hr 56°C) serum using Protein G HP SpinTrap columns (GE Healthcare) according to the manufacturer’s instructions. Eluted IgG fractions were quantified spectrophotometrically (Nanodrop). Neutralizing activity of IgG from immune rabbit sera was evaluated against primary isolates (Bx08, SF162, QH0692, MW965, 92BR025, DJ263.8, 92RW009) through the TZM-bl assay [37]. Briefly, 200 TCID_50_ of pseudovirus was incubated with four 2-fold dilutions of IgG from 250 μg/ml to 31.25 μg/ml for 1 h at 37°C in a total volume of 100 μl growth medium in 96-well flat-bottom culture plates. Freshly trypsinized cells (1 × 10^4^) were then added in 10% DMEM growth medium containing DEAE-dextran (Sigma) at a final concentration of 15 μg/ml. After 48 h incubation, 100 μl of growth medium was removed from each well and 100 μl of SteadyLite reagent (Perkin Elmer) was added. Luminescence was measured using a Berthold TriStar LB941 luminometer (PerkinElmer). The background controls contained cells only, while the virus controls contained cells plus virus. The percent neutralization for immune rabbit IgG was calculated comparing it to the virus controls. The 50% inhibitory dose (IC50) was calculated as the IgG concentration that induced a 50% reduction in RLU compared to the virus control wells, after subtraction of cell control RLU. Pre-bleed sera were used as negative controls. TriMab, a mix of 3 mAbs (b12, 2G12 and 2F5), (obtained from Centre for AIDS Reagents, NIBSC, UK) was used in every neutralization experiment as a strongly neutralizing control IgG.

## Results

### Production of soluble trimeric gp140_94UG018_

**Figure 1:**
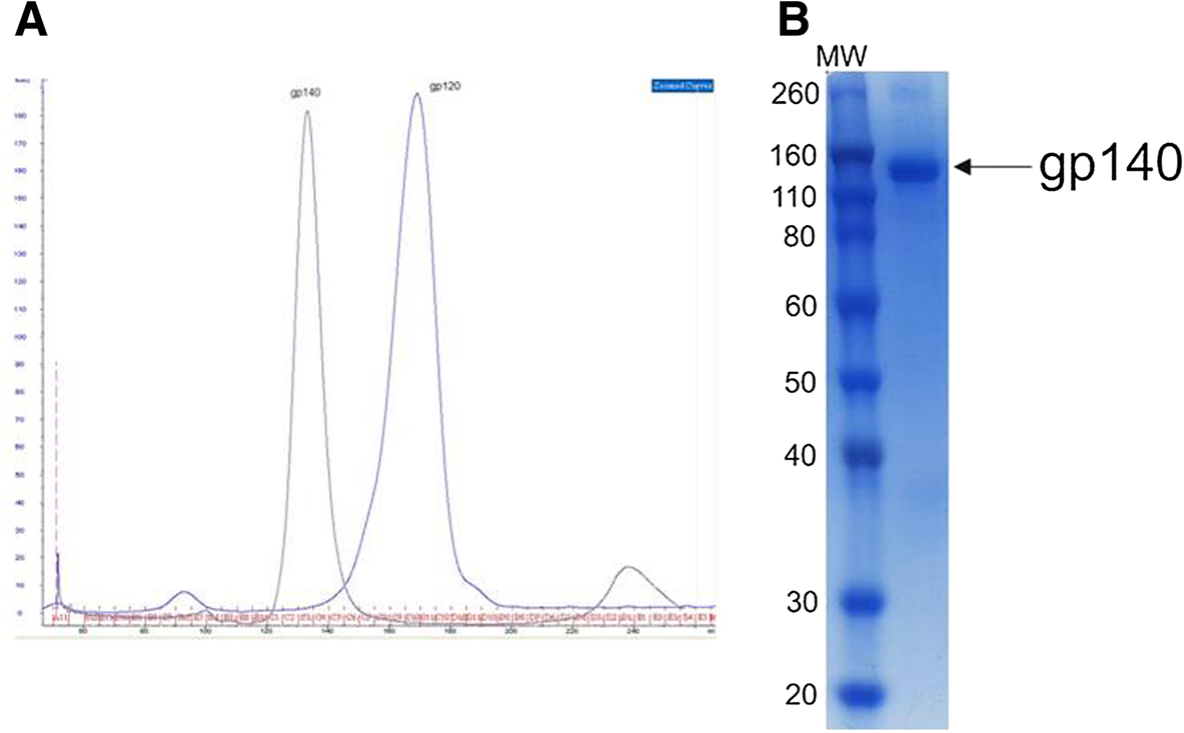
**Analysis of the gp140_94UG018_ protein. (A)** The trimeric form of recombinant gp140 protein was verified and purified by a size-exclusion chromatography. **(B)** Fractions corresponding to the gp140 trimer were pooled and analyzed by Coomassie stained denaturing SDS PAGE to confirm the purity of the product.

The recombinant gp140 protein was expressed at approximately 0.5 mg/liter of DMEM media from transiently transfected 293T cells, and protein expression was expanded to approximately 10 litres. Following a size-exclusion chromatography, fractions corresponding to the gp140 trimer were collected and pooled. Protein purity was analysed by SDS PAGE and shown to be >95% following the final size-exclusion step (Figure 1).

### Induction of anti-gp140 specific antibodies

**Figure 2:**
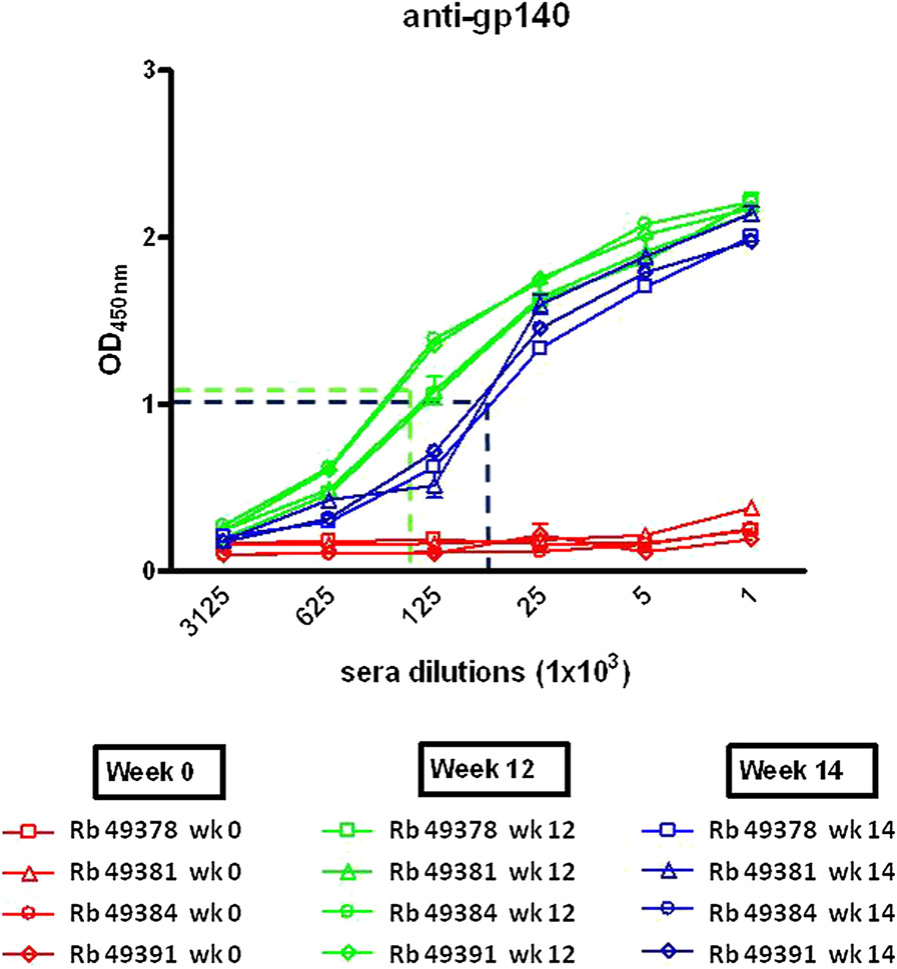
**Evaluation of IgG titers elicited in sera of immunized rabbits against the homologous trimeric gp140_94UG018_.** Five-fold dilutions of heat inactivated sera from pre-immunized (week 0) and immunized rabbits (week 12 and 14) were evaluated in ELISA for their reactivity with homologous gp140. Week 12 and 14 correspond to 4 and 6 weeks after the last antigen administration. The 50% binding is indicated for the week 12 and 14 sera.

Induction of a humoral immune response by soluble trimeric Clade A gp140_94UG018_ was evaluated in sera by ELISA performed on microwell plates coated with the monomeric form of the protein used for immunization protocol. Pre-immunization, week 12 and week 14 sera were tested and specific anti-gp140 antibodies were identified in sera from immunized rabbits, with a response stronger at week 12 than at week 14 (Figure 2). Indeed, all four sera from immunized animals at week 12 show a binding to the gp140_94UG018_, at the 1:625,000 dilution, statistically significant higher than pre-immunized sera (week 0) (p < 0.01). On the contrary, at the same serum dilution, only one (Rb 49381) of the immunized sera at week 14 show a binding statistically significant higher than pre-immunized sera (week 0) (p < 0.05). Moreover, a 50% of maximal binding activity was obtained, on average, with a 1:2.25 × 10^5^ dilution of week 12 serum and a 1:6.5 × 10^4^ dilution of the week 14 serum (p < 0.01), indicating the elicitation of a strong anti-gp140 antibody response four to six weeks after last immunization. Pre-immunization sera showed no positive reactions at any of the dilutions tested.

### Determination of broadness of humoral immune response to Env

**Figure 3:**
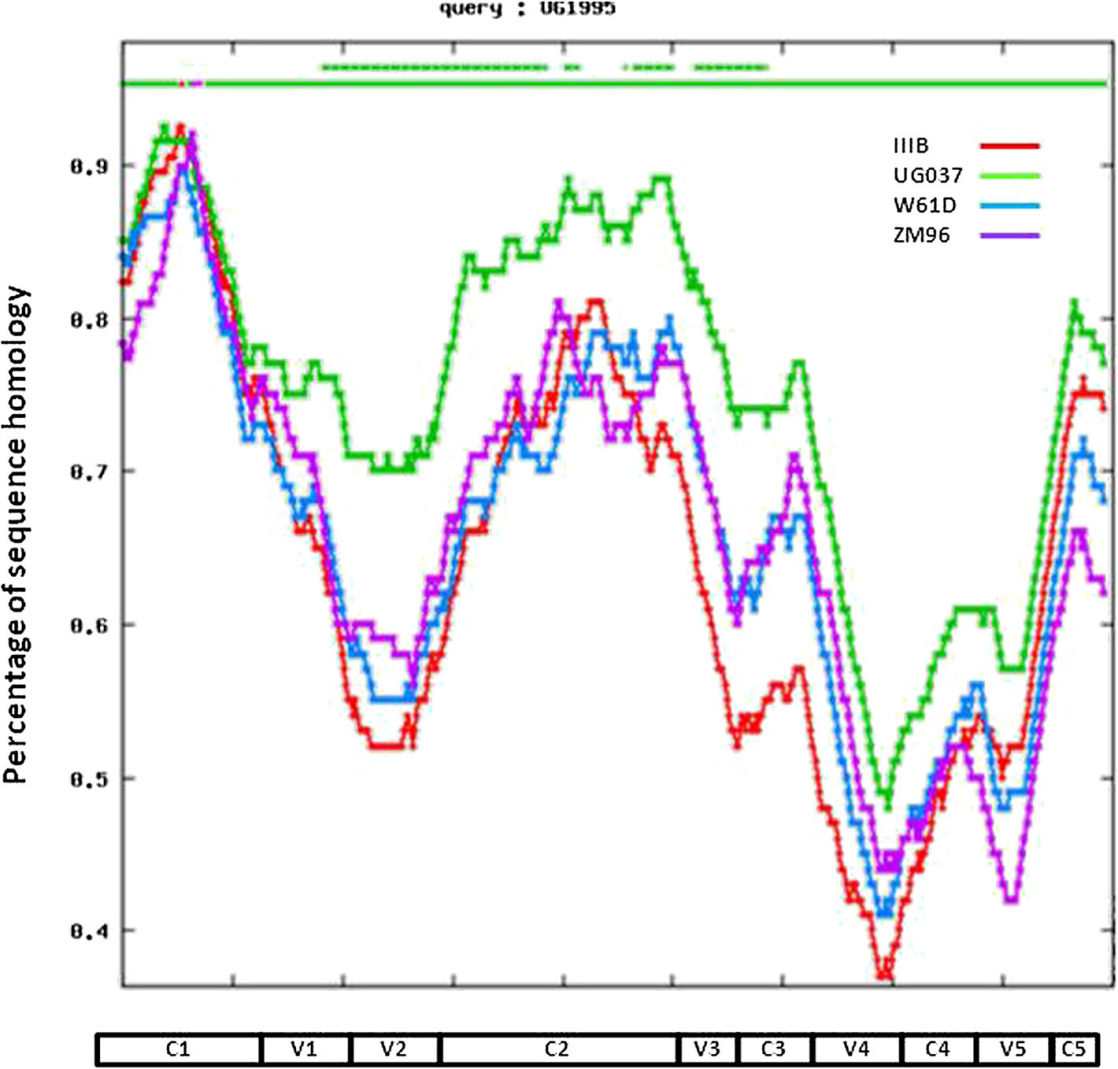
**Analysis of sequence homology.** The gp120 amino acid sequence of the indicated HIV isolates were aligned with the 94UG018 clade A Ugandan isolate. Sequence homology between each isolate vs the 94UG018 isolate is indicated along the whole gp120 sequence.

In order to evaluate the broadness of the binding characterizing the sera from animals immunized with the trimeric gp140_94UG018_, pre-immune (week 0) and immune sera (week 12) were tested in ELISA against the recombinant HIV gp120/gp140 proteins of different clades, i.e. IIIB (Clade B), W61D (Clade B), ZM96 (Clade C) and UG037 (Clade A). These proteins show, along the entire gp120 sequence, a pattern of similarity with our Ugandan glycoprotein in particular in the constant regions (i.e. C1, C2 and C5) and significantly lower in the variable regions (Figure 3). This is observed also for the UG037, which is of the same clade (Figure 3).

**Figure 4:**
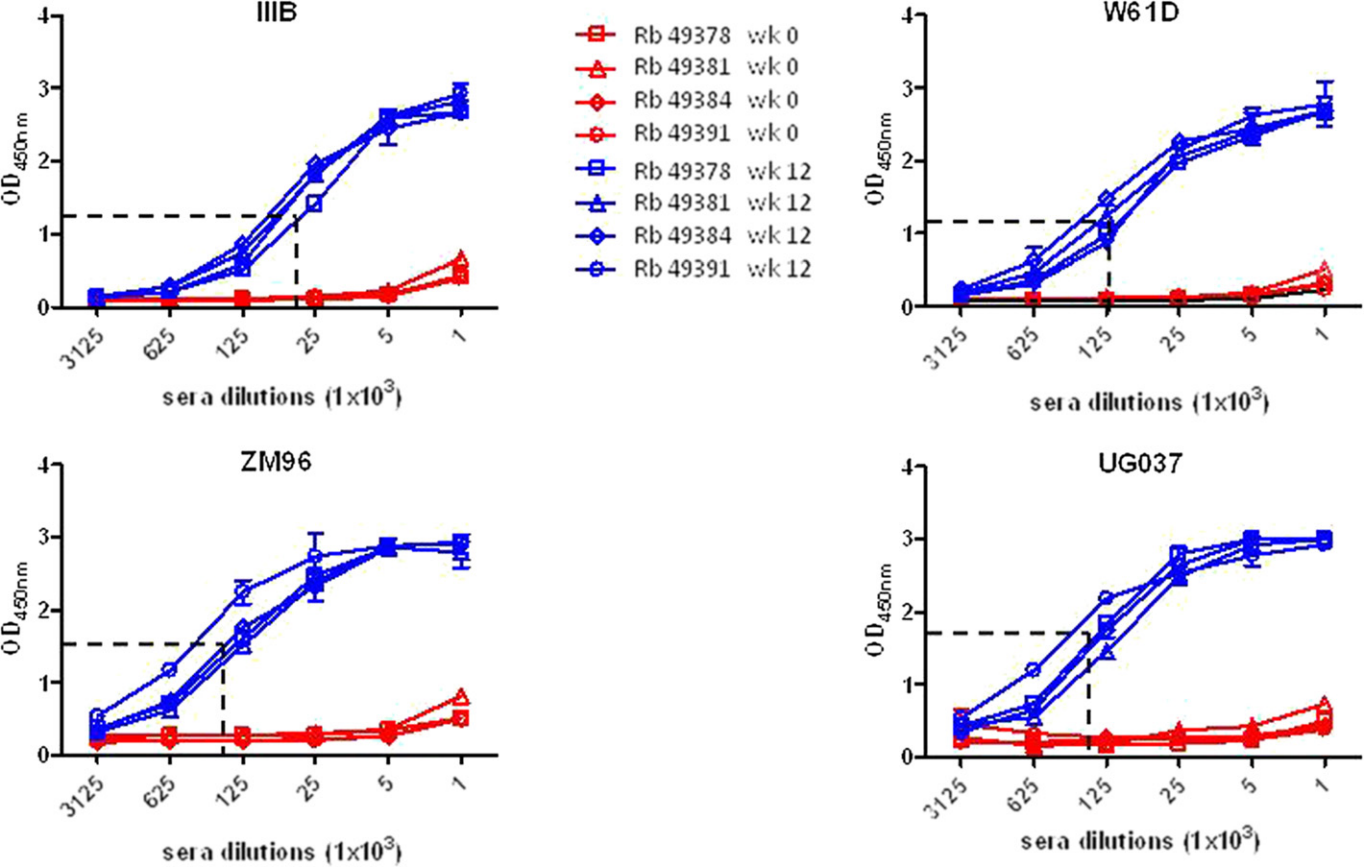
**Evaluation of IgG titers elicited in sera of immunized rabbits against heterologous HIV gp120.** Five-fold dilutions of heat inactivated rabbits sera, collected at 4 weeks after the last antigen administration (week 12), were evaluated in ELISA for their reactivity with heterologous gp120s. The 50% binding is indicated.

Sera from all immunized animals showed a high antibody titer at week 12 against all tested proteins, with a ranking of UG037 = ZM96 > W61D > IIIB (p < 0.01) (Figure 4). In particular, results show that 50% binding activity was obtained, on average, with a 1:2.25 × 10^5^ dilution for the UG037 and ZM96 proteins, exactly the same as for the homologous 94UG018 protein (Figure 2), a 1:1.25 × 10^5^ dilution for the W61D protein and a 1:4.5 × 10^4^ dilution for the IIIB protein (Figure 4). The ELISA results clearly show that immunization with the trimeric gp140_94UG018_ induces an immune response with a broad binding activity, recognizing HIV gp120 molecules from different clades.

### Evaluation of envelope epitopes recognized by the immune sera

**Figure 5:**
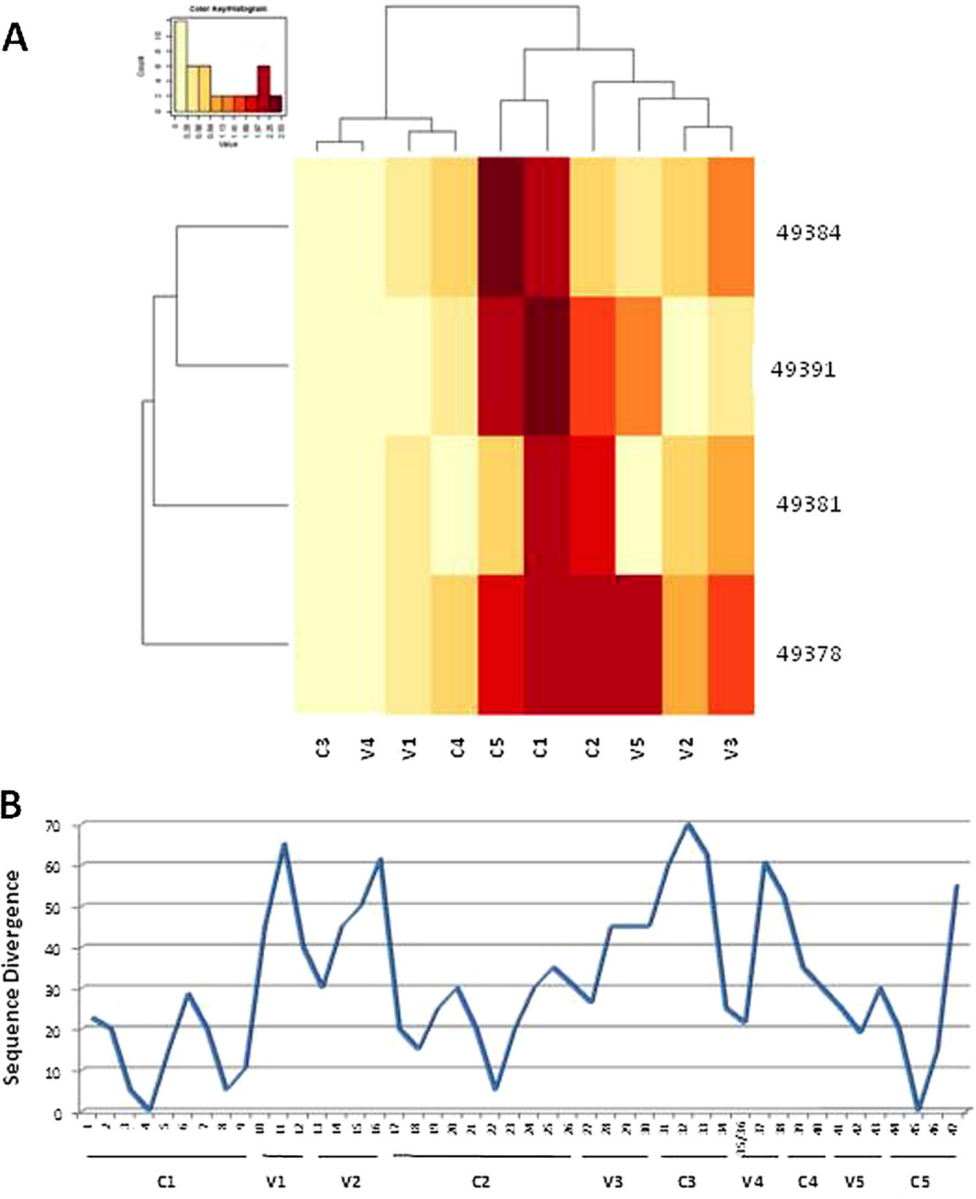
**Heatmap of binding to gp120 peptides’ pool. (A)** Intensity of serum binding in ELISA to peptides’ pool covering the constant and variable regions of gp120 is shown as heatmap. Increasing O.D. value, indicating stronger binding, is represented as darker color. **(B)** Sequence divergence between peptides used as target in ELISA and the 94UG018 clade A Ugandan isolate is indicated along the whole gp120 sequence.

In order to identify the main gp120 epitopes recognized by the immune sera elicited by the trimeric gp140_94UG018_, epitope mapping was carried out using 20 mer overlapping synthetic peptides spanning the entire length of gp120 from the C1 to the C5 region. A tiled array of 47 synthetic peptides (20 aa each) overlapping by 10 residues covering the entire length of gp120 (Table 1) was used for epitope mapping. The heatmap is derived on the O.D._450_ values obtained at ELISA reacting sera from each animal to peptides: the higher is the O.D. value (e.g. higher affinity) the darker is the color in the heatmap. Results, showed that the 12 week sera from all immunized animals bound the C1, C2 and C5 constant regions with high affinity and the V2, V3 and V5 variable regions with lower affinity (Figure 5A). The remaining C3 and C4 constant regions as well as the V1 and V4 variable regions were bound with limited affinity or not bound at all. These results strongly correlated with the percentage of divergence between the sequence of the gp140_94UG018_ and the peptides used as targets in the ELISA (Figure 5B). Nevertheless, it is relevant to note that the immunized animals showed a significant breadth of binding to the target epitopes, suggesting that the same vaccine molecule is able to elicit distinct patterns of antibodies focusing on different epitopes.

**Table 1:** Inhibitory concentration (IC) 50 for each animal to each viral isolate evaluated in the study

**Virus**	**Week 12**				**Week 14**			
	***49378***	***49381***	***49384***	***49391***	***49378***	***49381***	***49384***	***49391***
**SF162**	62.5	31.25	62.5	31.25	125	125	125	125
**BX08**	62.5	62.5	125	31.25	250	62.5	>250	>250
**MW965**	62.5	62.5	62.5	31.25	125	125	125	31.25
**DJ263.8**	>250	>250	>250	125	250	>250	>250	250
**92Br025**	>250	>250	>250	>250	>250	>250	>250	>250
**92RW009**	>250	>250	>250	>250	>250	>250	>250	>250
**QH0692**	>250	>250	>250	>250	>250	>250	>250	>250

Values are expressed in μg/ml of IgG and represent the reciprocal dilutions.

**Figure 6:**
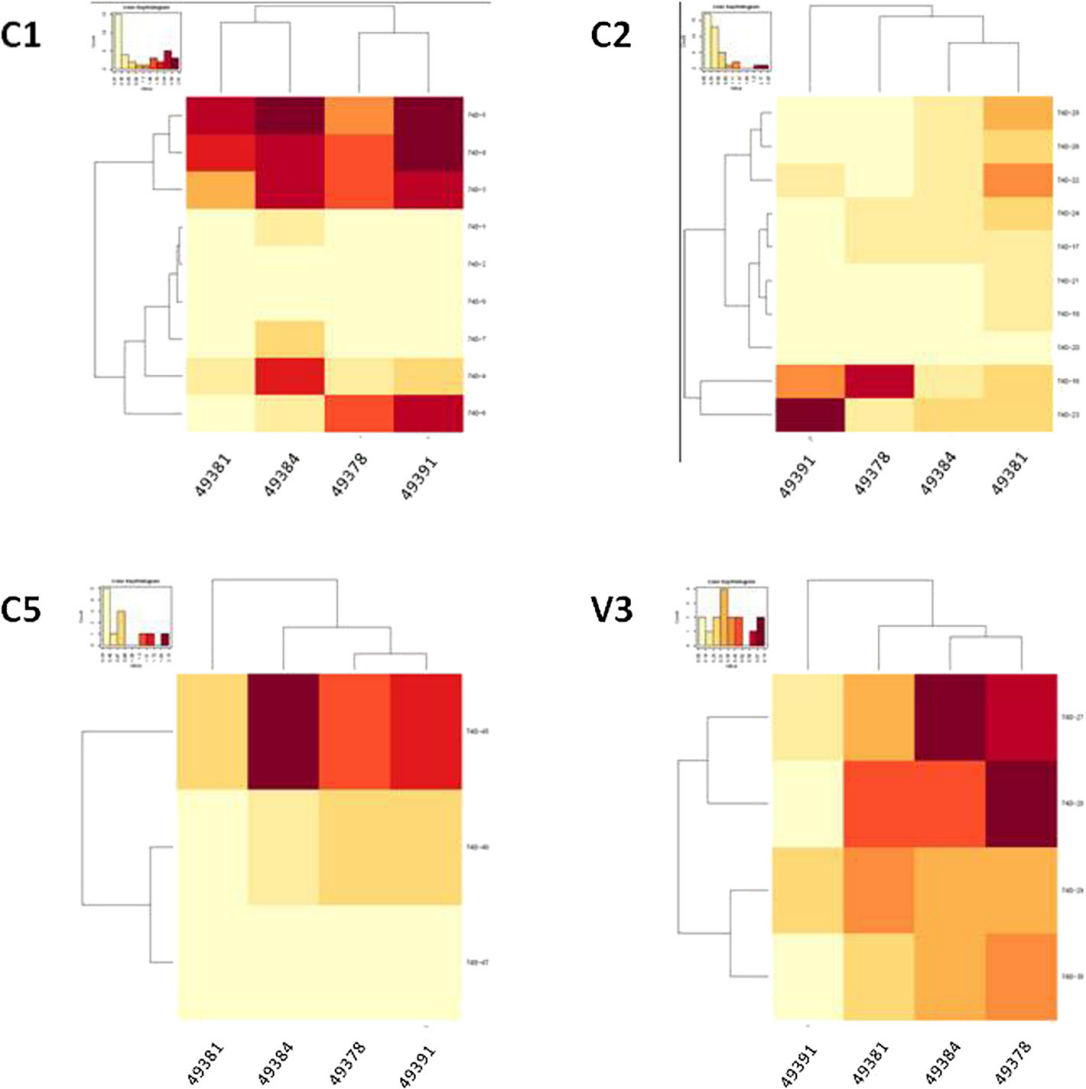
**Heatmap of binding to individual gp120 peptides.** Intensity of serum binding in ELISA to each overlapping peptide covering the indicated regions of gp120 is shown as heatmap. In each panel stronger binding is represented as darker color.

Within the overlapping peptide pools with the highest binding responses, individual overlapping peptides were used as targets for ELISA using sera collected at week 12 (Table 2). The heatmap built on the O.D._450_ values shows that, for each rabbit serum, reactivity against individual peptides was not homogeneous, indicating that the reactivity observed against the pools were attributable to specific epitopes (Figure 6). Moreover, most peptides were recognized by each rabbit serum with a broad range of binding efficiency, suggesting that specific regions of the gp140_94UG018_ have different potency in eliciting an immune response in the evaluated animals (Figure 5 and Table 2) [38]. The regions best bound by antibodies from immunized sera are depicted in an X-ray conformational structure of gp120 (Additional file 1: Figure S1).

**Table 2:** Overlapping peptides from HIV_IIIB_ used to fine mapping the binding of rabbit sera elicited by the trimeric gp140_Uganda_

**Gp120 region**	**Peptide code**	**Sequence**	**Gp120 region**	**Peptide code**	**Sequence**
C1	ARP740-1	ATEKLWVTVYYGVPVWKEATTT	C2	ARP740-24	NGSLAEEEVVIRSVNFTDNA
	ARP740-2	VPVWKEATTTLFCASDAKAY		ARP740-25	IRSVNFTDNAKTIIVQLNTS
	**ARP740-3**	**LFCASDAKAYDTEVHNVWAT**		ARP740-26	VQLNTSVEINCTR
	ARP740-4	DTEVHNVWATHACVPTDPN	V3	**ARP740-27**	**VEINCTRPNNNTRKRIRIQ**
	**ARP740-5**	**HACVPTDPNPQEVVLVNVTE**		**ARP740-28**	**NTRKRIRIQRGPGRAFVTIG**
	ARP740-6	PQEVVLVNVTENFDMWKNDMV		ARP740-29	RGPGRAFVTIGKIGNMRQA
	ARP740-7	NFDMWKNDMVEQMHEDIISL		ARP740-30	KIGNMRQAHCNISRAKWNNT
	**ARP740-8**	**EQMHEDIISLWDQSLKPCVK**	C3	ARP740-31	HCNISRAKWNNTLKQIDSKL
	ARP740-9	WDQSLKPCVKLTPLCVSLK		ARP740-32	LKQIDSKLREQFGNNKTIIF
V1	ARP740-10	LTPLCVSLKCTDLKNDTNTN		ARP740-33	REQFGNNKTIIFKQSSGGDPE
	ARP740-11	CTDLKNDTNTNSSSGRMIMEK		ARP740-34	KQSSGGDPEIVTHSFNCGGE
	ARP740-12	SSSGRMIMEKGEIKNCSFNI	V4	ARP740-35/36	GEFFYCNSTQLFNS
V2	ARP740-13	GEIKNCSFNISTSIRGKVQK		ARP740-37	NSTWFNSTWSTEGSNNTEGS
	ARP740-14	STSIRGKVQKEYAFFYKLDI		ARP740-38	TEGSNNTEGSDTTTLPCRI
	ARP740-15	EYAFFYKLDIIPIDNDTTSY	C4	ARP740-39	DTTTLPCRIKQIINMWQKVG
	ARP740-16	IPIDNDTTSYSLTSCNTSVI		ARP740-40	KQIINMWQKVGKAMYAPPIS
	ARP740-17	SLTSCNTSVITQACPKVSFE	V5	ARP740-41	KAMYAPPISGQIRCSSNITG
	**ARP740-18**	**TQACPKVSFEPIPHYCAPA**		ARP740-42	GQIRCSSNITGLLLTRDGGNS
	ARP740-19	PIPHYCAPAGFAILKCNNK		ARP740-43	LLLTRDGGNSNNESEIFRLG
C2	ARP740-20	GFAILKCNNKTFNGTGPCNT	C5	ARP740-44	NNESEIFRLGGGDMRDNWRS
	ARP740-21	TFNGTGPCNTVSTVQCTHGI		**ARP740-45**	**GGDMRDNWRSELYKYKVVKI**
	ARP740-22	VSTVQCTHGIRPVVSTQLLL		ARP740-46	ELYKYKVVKIEPLGVAPTKA
	**ARP740-23**	**RPVVSTQLLLNGSLAEEEVV**		ARP740-47	EPLGVAPTKAKRRVVQREKR

Bold characters indicate the peptide with the strongest binding by immune sera.

### Breadth of neutralizing antibodies (NAbs) elicited by vaccination with recombinant trimeric gp140_94UG018_

**Figure 7:**
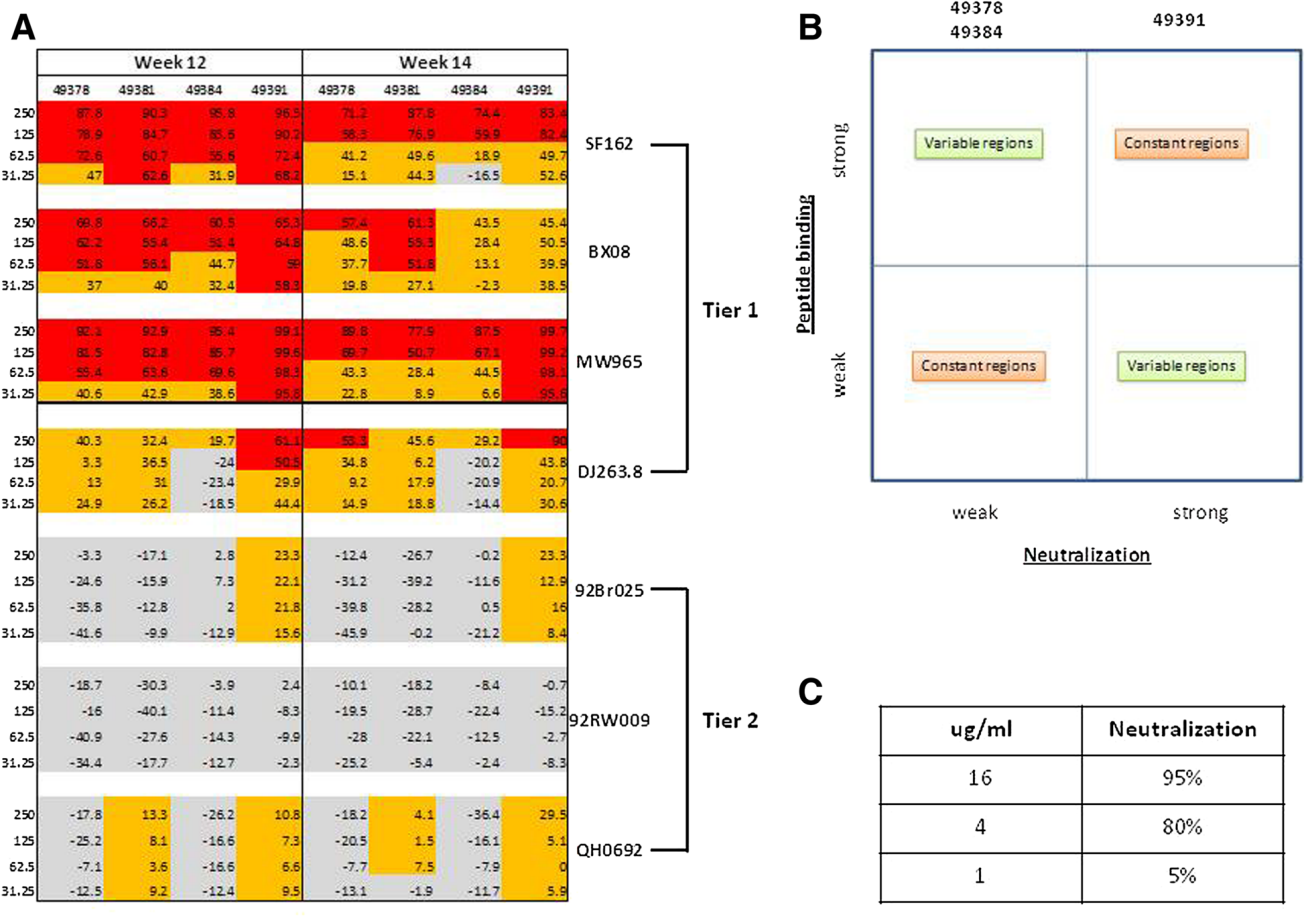
**Neutralization activity of sera from immunized rabbits. (A)** Neutralization activity in TZM-bl cells obtained with serial dilutions of IgG (expressed as μg/ml) purified from immunized sera is shown as heatmap for each virus tested. Neutralization activity >50% and <50% is indicated in red and orange, respectively, whereas absence of neutralization activity is indicated in grey. Pre-bleed sera showed a negligible neutralization activity (<10%). **(B)** Correlation matrix between neutralization activity and peptide binding to envelope gp120 regions. **(C)** Neutralization activity of TriMab.

Induction of Nabs in rabbits immunized with recombinant trimeric gp140_94UG018_ was evaluated in the TZM-bl neutralization assay against a panel of 7 HIV-1 isolates using IgG purified from sera collected at week 0, 12 and 14 (T_0_, T_12_ and T_14_ respectively). In particular, 3 viruses were clade B isolates (Bx08, SF162 and QH0692), 2 were clade C viruses (MW965 and 92BR025), 1 virus belonged to the 02_AG subtype (DJ263.8) and 1 virus was a clade A and C mosaic isolate (92RW009). The virus isolates MW965, 92Br025, DJ263.8, Bx08 and SF162 are known to be Tier 1 while the virus isolate 92RW009 and QH0692 are classified as Tier 2 isolates. A heatmap was generated based upon the 50% neutralization effect, indicating in red the neutralization results above 50% for each IgG concentration (Figure 7A).

The overall results showed that the immune sera were able to mainly neutralize tier 1 viruses across clades and that this neutralization potency waned over time. Considering the T_12_ sera, 3 of the 5 Tier 1 virus isolates (SF162, Bx08 and MW965) were neutralized with an IC_50_ of 62.5 μg/ml by all sera and with an IC_50_ of 31.25 μg/ml by serum from the 49391 rabbit. The latter serum showed a neutralization activity also against DJ263.8 with an IC_50_ of 125 μg/ml (Figure 7A and Table 1), while no neutralization against 92Br025 could be detected. Tier 2 viruses were poorly or not neutralized in all cases.

Comparing the breadth and potency of neutralization, it is noteworthy that the best performing serum (49391) showed the strongest binding to the constant regions (p <0.05) and the lowest binding to the variable regions (V2 and V3) (p < 0.001), compared to the other sera. On the contrary, the least performing sera (49378 and 49384) show a significant binding to the constant regions, but the strongest binding to the V2 and V3 variable regions (p < 0.01) (Figure 7B).

## Discussion

In this study we evaluated the immunogenicity of a soluble trimeric gp140_94UG018_ derived from a clade A HIV-1 isolate administrated subcutaneously to rabbits. All four immunized animals developed high titers of specific anti-gp140 antibodies against the homologous gp140_94UG018_ protein, as well as against heterologous envelope glycoproteins of clade B (gp120_IIIB_ and gp120_W61D_), clade A (gp140_UG037_) and clade C (gp140_ZM96_) which present a significant sequence divergence compared to the gp140_94UG018_. The highest binding activity was observed against the two gp140 UG037 and ZM96 molecules, effect that could be due to the highest sequence homology to the gp140 94UG018 molecule (UG037) and/or the presence of the ectodomain of gp41 which is substantially conserved across the clades (UG037 and ZM96). Overall, these results indicate that although rabbits were immunized with a clade A derived gp140, this immunization was able to elicit a binding activity to cross-clade Env antigens. This result was supported by the relevant sequence homology between the different Env molecules, especially in the constant regions.

All immunized animals’ sera were able to bind the C1, C2 and C5 regions with high affinity and the V2, V3 and V5 regions with lower affinity, presenting a direct correlation with the percentage of divergence between the gp140_94UG018_ immunogen and the peptides used as target in the binding assay. However, the differential binding efficacy to the same peptide by different sera is highly suggestive of distinct patterns of immune response elicited by the same gp140_94UG018_ in outbred animals, indicating that the specificity of individual responses to the same vaccine antigen is not totally predictable. A more in-depth epitope mapping analysis performed with individual epitopes covering the entire length of gp120 from the C1 to the C5 region confirmed the potency ranking observed with the peptide pools but showed that, for each region, specific peptides are recognized more efficiently than others. This further supports the concept that the immunogen is able to elicit distinct patterns of Abs focused on different epitopes. Further analysis have been planned to be conducted using peptides based on the sequence of 94UG018 protein, to verify whether additional Ab specificities are identified in immune sera elicited by the gp140_94UG018_.

Sera from animals immunized with gp140_94UG018_ showed a >50% neutralization efficacy against 3 out of 5 Tier 1 pseudoviruses, whereas poor or no neutralization was observed against Tier 2 pseudoviruses.

This soluble trimeric clade A gp140_94UG018_ was able to induce a cross-clade neutralizing activity as demonstrated by the ability of the immune sera to neutralize Tier 1 pseudoviruses of different clades. Although the neutralization activity was limited to Tier 1 pseudoviruses, this result is in agreement with other immunogenicity studies performed with soluble HIV Envelope proteins of different clades with our data even suggesting improved neutralization in several instances [39-42]. Moreover, it is prospected to re-evaluate the breadth of neutralization activity in A3R5 cells, which have been recently shown to be more sensitive to neutralization than the TZM-bl used in the present study [43]. In particular, serum from the 49391 rabbit was the only one to show neutralization activity against four out of five Tier 1 with the highest activity and also to show very limited neutralization activity, although lower than the 50% threshold, against two of the Tier 2 pseudoviruses. This broader neutralization activity does not seem to be attributable to antibodies to the V3 domain, as serum from the 49391 rabbit, compared to the other sera, presents the weakest binding efficacy to V3 epitopes, considered both as pool or individual peptides. This observation is in contrast to previously reported data showing that broadly neutralizing activity, in sera from animals immunized with trimeric Clade A envelope molecules, is mostly associated to antibodies directed toward the envelope variable regions (V1, V2, V3) [44]. Interestingly, our gp140_94UG018_ seems to divert the immune response from variable region of envelope molecules. Further analyses will need to be performed since these results could be due to the high sequence divergence between the gp140_94UG018_ and the gp120_IIIB_ peptides used in our assay.

Of note, all four immunized sera are characterized by a stronger binding efficacy to epitopes of the constant and V5 regions, which are known to be involved in the CD4bs of the HIV gp120 and are targets of few broadly cross-clade neutralizing monoclonal antibodies (bnAbs) [11,45,46]. The poor or absent binding to C3 epitopes is most likely due to the extremely high divergence (>60%) of the C3 region sequence in the gp140_94UG018_ which may severely affect the Ab-epitope recognition. Nevertheless, the strong binding to epitopes covering regions involved in the CD4bs may possibly suggest that such antibodies may play a relevant role in the neutralization activity of immunized sera, although the broadness of their activity is confined only to Tier 1 isolates. Indeed, the observed strong binding to C1 epitopes may possibly play a role in the insufficient pattern of neutralization, given that antibodies binding to C1-C4 domains have been reported to compete with CD4bs broadly cross-neutralizing antibodies for binding [47]. Moreover, Abs specific to the C1 region have been reported to be associated with induction of antibody-dependent cellular cytotoxicity (ADCC) [48], which plays a role in protection from HIV infection and disease progression, as shown also in the RV144 Thai vaccine trial [49-51]. Overall, the results of the present study highlight that trimeric clade A gp140_94UG018_ is a very effective immunogen capable of inducing significant cross-clade humoral immune responses in the rabbit model. The gp120 epitope mapping provides potential relevant insights to clarify the neutralization activity of the elicited immune sera confined to Tier 1 isolates. According to these observations, possible structural modifications of the clade A gp140_94UG018_ can be envisaged (i.e. deletion of the C1 region) to improve the breadth of the neutralization activity.

Our results provide a rationale for the design and evaluation of immunogens to be used in HIV vaccine strategies. In particular, clade A gp140_94UG018_ shows promising characteristics for potential involvement in an effective HIV immunization regimen.

## Competing interests

The authors declare that they have no competing interests of either financial or non-financial nature regarding the work described in the present manuscript and its publication.

## Authors’ contributions

All authors conceived and designed the experiments. Performed the experiments: MLV, MT, LH, MJ, BG. Analyzed the data: GV, AF, GS, LB. Contributed reagents/materials/analysis tools: GS-J, MR. Wrote the paper: MLV, MT, LB. All authors complemented and approved the final version of the manuscript.

## Supplementary Material

Additional file 1: Figure S1 Display of the regions bound in the gp120 sequences. The conformational structure of the indicated regions in the gp120 sequence, which are best recognized by sera from immunized rabbits, is shown in red. Conformation of the gp120 context as revealed by the X-ray structure (PDB code 2QAD) was used as a prototype to identify variable and constant regions best bound by antibodies of immunized sera [52]. Click here for file
