# Supplementary figures and images for: Characterization of humoral responses to soluble trimeric HIV gp140 from a clade A Ugandan field isolate
Source: J Transl Med. 2013 Jul 8;11:165. doi: 10.1186/1479-5876-11-165 (PMC3729709; doi:10.1186/1479-5876-11-165)

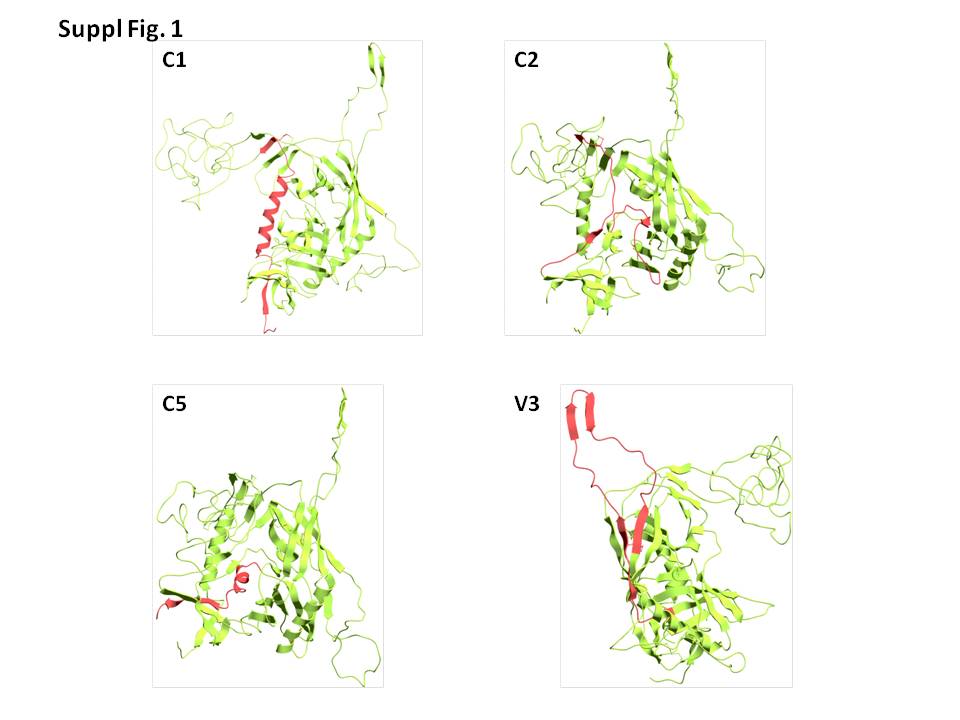

Supplement: Additional file 1: Figure S1 — Display of the regions bound in the gp120 sequences. The conformational structure of the indicated regions in the gp120 sequence, which are best recognized by sera from immunized rabbits, is shown in red. Conformation of the gp120 context as revealed by the X-ray structure (PDB code 2QAD) was used as a prototype to identify variable and constant regions best bound by antibodies of immunized sera [52]. [file 1479-5876-11-165-S1.jpeg]
